# Abstract of Proceedings of the Buffalo Medical Association

**Published:** 1867-06

**Authors:** T. M. Johnson

**Affiliations:** Sec’y.


BUFFALO

### Medical and Surgical Journal.

VOL. VI. JUNE, 1867. No. 11.

### Original Communications.

ART. I.—*Abstract of Proceedings of the Buffalo Medical Association.*

TUESDAY EVENING, May 7th, 1867.

The meeting was called to order by the President. Members present—Drs. Gould, Eastman, Little, Lothrop, Wetmore, Samo, Camerling, Gay, Nichell, Daggett, Sheldon, Schuyler, Strong and Johnson.

The minutes of the last meeting were read and approved.

DR. STRONG moved that the regular order of business be suspended that the Association may hear the valedictory address of Dr. Gould, the retiring President. Carried.

DR. GOULD then read the following valedictory address:

GENTLEMEN:—It is an established custom for the retiring President to address a few parting words to the members of the Association, a custom perhaps more honored in the breach than observance. But, as the precedent is established, I must follow in the footsteps of those who have gone before, and give you my parting benediction.

The occasion does not seem to me to require a dissertation upon any special subject, but more appropriately a review of the condition and doings of the Association during the past year. This, with some allusions and comparisons of ancient sanitary customs with those of our own day, and a glance at our losses, during the past ten years, will comprise the main points upon which I propose to occupy a few moments of your time.

Our worthy and efficient Treasurer reports the Association free from debt, and a small balance in his hands. As he makes a full statement of the finances, I do not deem it necessary to dwell upon the subject. Yet there is one point in this connection that I would urge upon the attention of the members, and that is, the prompt payment of the yearly dues. I can recognize no valid reason why members should allow themselves to be one, two, or three years in arrears for dues.

I regret to notice the fact, that during the past year, our meetings have not been as well attended as they should have been. Whether this is to be ascribed to indifference, forgetfulness, or circumstances beyond control, it may not become me to say. On a former occasion I took the liberty of calling attention to this matter, and suggested as a remedy, more frequent meetings. Often during the past year we have had a bare quorum present, and more than once our meeting went by default. With a membership of forty or more, this should not be, and for the credit of the profession in as large a city as this, an Association like ours, should not be as it has been. The oldest and most experienced members may derive pleasure and profit from mutual interchange of thought, and the advancement of new ideas, and certainly the young and inexperienced members can always learn therefrom something new and useful. Negligent, as I have been in years past, I can say with truth, that I never attended a meeting of this Association that I did not learn something, that sooner or later, was of practical benefit—the history of a case, the discovery of some new remedy, or improvement in the treatment of some disease by some of the more experienced of my professional brethren, always proved to me both profitable and instructive. For this reason alone, would I urge upon all, a more strict attendance in the future, and a more general custom of reporting cases. If a case is successfully treated by old and established remedies, let us know it, for it is good news. If a new and improved treatment is tried, give us the result, for that too will be good news, whether it proves successful or not. Let every one report something, and thereby help to increase the interest of our meetings. Too many think they cannot report cases that will interest the members of the Association, and also fear that their views may be criticised, or their treatment condemned by older members. To those let me say, do not fear that, for if wrong, a fair and honest discussion of the subject, will in the end benefit more than it will injure, even if not approved. Then, again, let the young men remember that the older and now most active members, will necessarily soon pass away; must in due course of time vacate the stage, and their places be filled by those younger in years and in practice. Therefore, let me say, hide not your talent, but come boldly up to the mark, and let your light shine. Be not only attendants, but participants in the good work.

The revival during the winter of the old plan of having a dissertation read on some special subject, by a member elected at a previous meeting, is well calculated to elicit debate, and if continued in a proper manner and spirit, will greatly add to the usefulness and interest of the meetings of the Association.

On assuming the duties of the chair, I took the liberty of speaking upon matters pertaining to the general sanitary condition of our city in view of the impending danger of a visitation of Asiatic cholera. Providentially, we were spared, only perhaps for a season. Fortunately for us, the past year has been one of unusual freedom from any serious epidemic disease. However, all I then said on the subject will apply with equal force at the present time. I do not believe that the Board of Health, as at present organized, will prove much superior to that of the past. As long as commissioners are selected, on account merely of political expediency, we may reasonably expect them to understand political "*tactics*" much better than mortuary statistics.

It has been said, and probably with truth in some respects, that if the people of ancient times could re-visit the earth, they would be greatly surprised. No doubt they would, in some things. But in regard to sanitary improvements, I verily believe the laugh would be against us. For instance, Rome two thousand years ago, had her eight hundred public baths, hot, cold and vapor, and what was better still, the people, rich and poor, used them freely. With us, the lake and canals afford the only bathing accommodation for the poor, and if they go there, they subject themselves very properly to arrest and punishment. No people of modern times have equaled the Romans in the magnificence of the works which they constructed for the purpose of bringing supplies of water to their provincial towns, as well as to Rome itself. Such a quantity of water was introduced into the city, that whole rivers seemed to flow through the streets and down the sewers, so that every house had its pipes and cisterns, sufficient to furnish a copious and abundant supply. Their aqueducts are incontestible monuments of the greatness of their designs, and valleys, mountains and extensive plains, offered no impediment which they did not surmount by skill, and the exercise of an indomitable will.

It is said that Agrippa alone erected one hundred and thirty reservoirs, and opened one hundred and five fountains in connection with them. The daily supply of water amounted to eight hundred thousand tons. The quality was extremely salubrious and the quantity abundant. The arrangements for an equal distribution were on a scale of convenience as well as magnificence. Every quarter of the city, however poor, was equally well supplied. From the sight of that magnificent fountain of Paul V, at Rome, bring one of her citizens and place him beside our fountain in the Court House Park, and tell him that he has traveled down the long vista, from the dark ages of antiquity, to the year of our Lord 1867, the age of great improvements, and that the monument before him was one of the evidences of progress, and on which side, think you, would the laugh be?

The Romans, regardless of labor or expense, built their aqueducts over valleys, supported by thousands of arches, and tunneled through solid rock for miles, by which means each inhabitant was supplied with ten times the amount of pure water that we are supplied with.

Our water works consist of a reservoir made of clay; an engine house, and a short subterranean canal or tunnel under the pier to the edge of the river, from which for about four months in the year we are supplied with water enriched with the aroma, if not the more solid contents of our sewers; all of which could be easily avoided by a few yards of tunneling, which would give us the pure water of lake Erie the whole year, as any one can see by observing what a short distance the water in the river is affected beyond the pier. The city and private citizens pay enormously for the privilege of swallowing the nauseous dose one-third of the year.

In our sister city of Chicago, the people became disgusted with the water taken from the edge of the lake, which, like our Niagara, contained too many rich things finely flavored from the city sewerage, and have lately penetrated under the lake two miles, and her citizens are now supplied with pure, clean water, "sweet as the stream that gushes from the mountain side."

An abundant supply of pure water was a special characteristic of the ancients, and such an abuse of the laws of health, as we are obliged to submit to in this respect, would not have been tolerated for a moment, when the remedy was so easy of accomplishment. In the laying out of all our great cities, this great natural want and sanitary requirement, is not thought of, and in every case is left for after consideration.

In the great city of London, the water is turned on only for a fraction of an hour in a day, and has to be received in cisterns, or barrels, consequently pure water is never at the Londoner's command. These receptacles, though filled every day, are seldom emptied, save on rare occasions, so that the water sent in weeks, months or years ago, though diluted by daily accessions, is never exhausted, and they go on drinking a mixture which has been imbibing impurities from a foul atmosphere, it is impossible to say how long, and the result upon the health of the community needs only to be suggested. On Sunday the water is not turned on at all, the consequence being, that on that day some half a million of the poor, who need it most, do not get any.

A writer in the *Times* of January last, says: "In many houses the water for the Sunday uses has to be begged from neighbors. Sometimes for part of Sunday and Monday, a whole court has to borrow for their scant necessities from a 'public' at the corner. Thus the day of all others, when the homes of the poor are crowded, the means of cleanliness and comfort are even less than on working days." Their own papers are our authority for what has been stated.

It is true that there has been discoveries and improvements in mechanism, and in the arts and sciences. And yet much that we boast of in that respect, are only re-discoveries of what had been lost during the dark ages, and were quite as well understood by the ancients three thousand years ago. In most that relates to sanitary laws, and the science of preserving health, we have been retrograding for ages. The great minds of the present day have been perverted, and turned into other channels. Inventions and accumulation are the order of the day, and this great question of human power, health and happiness, together with the laws which govern that power, is ignored, or almost entirely neglected. If any doubt my word, let them use their eyes. Let them visit our markets, tenement houses, etc., and there observe how half the people live, and on what they feed. But I must leave this subject, as I am trespassing too much on your time and good nature.

Thanks to a kind Providence, our ranks during the year, remain unbroken by death. This cannot always be. A few years and many familiar faces will be missed. My thoughts dwell sadly upon this, whenever I take a retrospective glance at the changes time has brought about since the organization of this Association.

Within the past ten years we have lost bright jewels and rough diamonds not a few. Permit me to mention in this connection the names of those who were then members, but who will meet with us no more. They rest where the midnight bell will no more arouse them from peaceful slumber. Beneath the waving branches of the cypress and the willow, they "sleep the sleep that knows no waking." Nobler, truer, trustier hearts, never beat in human breast. They rest from their labor, but their good works will live long after the hand that pens this tribute to their memory shall have mouldered away, and joined its kindred dust.

"We'll not forget them, we who stay
To toil a little longer here;
Their names, their faith, their worth shall lie
On memory's page, all bright and clear."

The record inscribed on memory's tablet, embrace the following names:

| | |
|---|---|
| Bissell, | Trowbridge, |
| Burwell, | Treat, |
| Lewis, | Wilcox, |
| Newman, | Lockwood, |
| Sprague, | Howell, |

Butler.

A loss in ten short years of nearly one-fourth of our members by death alone. Here we have a practical and positive illustration of the fact, that death seeks not alone the aged and infirm, for some of the number fell before arriving at middle age, while others had little more than reached the stage of manhood; others, however, had reached that stage when "the eye is dim and manly vigor abated." Thus we are taught that we are frail mortals, and know not what a day or an hour may bring forth. With the seeds of disease implanted in our nature, the food we eat, the water we drink, and the air we breathe, are all impregnated with death. All the promises of life are but dust. "They fade as the flower, and are as fleeting as the summer cloud."

During the same period we have lost by removal to other localities nineteen members, which would leave us twenty-nine out of fifty-eight, just half our membership ten years ago. To me there is a shade of sadness mingled with this retrospect of a single decade, for among those lost to us by death and removal were some whose warm personal friendship will cease to be remembered only with life itself. Taking a prospective view, what changes may we not reasonably expect in the same period to come? How many now present will meet in this room ten years from this evening? When that anniversary arrives, should I be spared, I hope to hear the retiring President, or some surviving member, make a similar statement of the change which time has wrought with the members of the Association.

Gentlemen, for your patience and attention to these rambling thoughts, and the kindness and consideration shown me on all occasions, espeecially while occupying the chair, and acting as your presiding officer, permit me to return you my sincere thanks.

At the conclusion of the address, Dr. Eastman moved that the thanks of the Association be tendered to Dr. Gould for his able and interesting address, and that a copy be requested for publication in the Journal. The vote was unanimous in the affirmative.

On motion of Dr. Gould, Drs. Strong and Gay, were appointed a committee to conduct the President to the chair.

Dr. Eastman, on taking the chair, thanked the Association for the honor conferred upon him in electing him President of the Association, and said that it was not until within a few hours that he had concluded to accept the position, and was not therefore prepared to make any extended remarks at this time, but hoped to be able to address them at a future meeting.

By vote of the Association Dr. Diehl was invited to attend the meetings until the next regular meeting of the Erie County Medical Society.

Reports of officers and committees being in order, the Secretary read a special report from the Treasurer, which was, by vote, referred to the auditing board.

Dr. Gay was elected to read an essay at the next meeting of the Association.

Adjourned. T. M. JOHNSON, Sec'y.

